# SEVERE PERTUSSIS IN CHILDHOOD: UPDATE AND CONTROVERSY - SYSTEMATIC REVIEW

**DOI:** 10.1590/1984-0462/;2019;37;3;00006

**Published:** 2019-05-16

**Authors:** Márcia Borges Machado, Saulo Duarte Passos

**Affiliations:** a Faculdade de Medicina de Jundiaí, Jundiaí, SP, Brazil.

**Keywords:** pertussis, Whooping cough, Macrolides, Antitussives agents, Leukocytosis, Decision making, pertussis, Coqueluche, Macrolídeos, Antitussígenos, Leucocitose, Tomada de decisões

## Abstract

**Objective:**

Through a systematic review, this essay aimed at revising the concepts of severe *pertussis*, updating the epidemiology, pathophysiology, clinical presentation, antibiotic therapy and auxiliary therapeutic options for symptomatology and complications.

**Data sources:**

This review considered publications from the last 30years in the databases US National Library of Medicine (PubMed), Scientific Electronic Library Online (SciELO), Literatura Latino-americana e do Caribe em Ciências da Saúde (LILACS), Cochrane, Google Scholar, as well as protocols of the Ministry of Health and recommendations of the Centers for Disease Control and Prevention, related to childhood *pertussis* (whooping cough), with emphasis on its severe form. This research was based on keywords derived from the terms “*pertussis*”, “azithromycin”, “antitussives”, “leukocyte reduction” in Portuguese and English. Duplicate studies and those with unavailable full-text were excluded.

**Data synthesis:**

Among 556 records found, 54 were selected for analysis. *Pertussis*, as a reemerging disease, has affected all age groups, evidencing the transient immunity conferred by infection and vaccination. Severe cases occur in neonates and infants, with secondary viral and bacterial complications and malignant *pertussis*, a longside hyperleukocytosis, respiratory failure and shock. Macrolides continue to be the chosen antibiotics, while antitussives for coughing remain without efficacy. The prompt treatment in Intensive Care Units improved the prognostic in severe cases, and transfusion was promising among procedures for leukoreduction.

**Conclusions:**

Approaching severe pertussis in childhood remains a challenge for diagnostic and therapy, as the available therapeutic options are still unsatisfactory. Strategies of prevention are expected to reduce the occurrence of severe cases, while new studies should confirm the role of auxiliary therapies.

## RESUMO

**Objetivo:**

Rever os conceitos de coqueluche grave, atualizar epidemiologia, fisiopatologia e apresentação clínica, verificar as recomendações de antibioticoterapia e conhecer opções terapêuticas auxiliares na sintomatologia e complicações, por meio de revisão sistemática.

**Fontes de dados:**

Foram pesquisados trabalhos publicados nos últimos 30 anos nas bases US National Library of Medicine (PubMed), Scientific Electronic Library Online (SciELO), Literatura Latino-americana e do Caribe em Ciências da Saúde (LILACS), Cochrane e Google Scholar, bem como protocolos do Ministério da Saúde e recomendações do Centers for Disease Control and Prevention, relacionados à coqueluche na infância, com ênfase na forma grave. Apesquisa baseou-se em palavras-chave derivadas dos termos “coqueluche”, “azitromicina”, “antitussígenos” e “redução de leucócitos”, nos idiomas português e inglês. Foramexcluídos estudos em duplicata ou texto integral indisponíveis.

**Síntese dos dados:**

Dos 556 registros encontrados, foram selecionados 54 para análise. A coqueluche, como doença reemergente, tem acometido todas as faixas etárias, evidenciando a imunidade transitória conferida pela infecção e pela vacinação. Quadros graves ocorrem em neonatos e lactentes, com complicações virais e bacterianas secundárias e *pertussis* maligna, com hiperleucocitose, insuficiência respiratória e choque refratário. Os macrolídeos continuam como antibióticos de escolha. Os sintomáticos da tosse não demonstraram eficácia. O suporte precoce em Unidade de Terapia Intensiva melhorou o prognóstico dos casos graves e a exsanguineotransfusão se mostrou a mais promissora entre os procedimentos para leucorredução.

**Conclusões:**

A abordagem da coqueluche grave na infância segue como desafio diagnóstico e terapêutico. As opções terapêuticas disponíveis ainda são insatisfatórias. Espera-se que as estratégias de prevenção reduzam a ocorrência de casos graves e que novos estudos confirmem o papel das terapias adjuvantes.

## INTRODUCTION

Pertussis is a common disease that affects all age groups. Youngchildren may develop severe complications such as apnea, cyanosis, pneumonia, pulmonary hypertension, respiratory failure, and seizures.^1^ ^,^ ^2^ Over the last decade, Brazil and the world were surprised by its increased incidence, especially in unvaccinated infants.^1^ ^,^ ^2^ ^,^ ^3^ Inadolescents and adults, pertussis (also known as whooping cough) had atypical clinical presentations, with infected mothers being the main source of transmission.^2^ ^,^ ^3^ ^,^ ^4^ Challenging for health professionals, pertussis’ epidemiological profile, diagnosis and treatment have gone through changes.^2^ ^,^ ^3^ ^,^ ^5^ The severe forms have received special attention, with encouragement of research on early support at Intensive Care Units (ICUs) and complementary treatments, such as exchange transfusion and potential therapies in experimentation; immunosuppressants and anionic modulators, still lacking consensus.^6^ ^,^ ^7^ Thus,the aim of this article was to review concepts of severe pertussis, to update information about its epidemiology, clinical presentation, diagnosis, antibiotic therapy, symptomatic therapeutic options and complications, by means of a systematic review.

## METHOD

**Figure 1:**
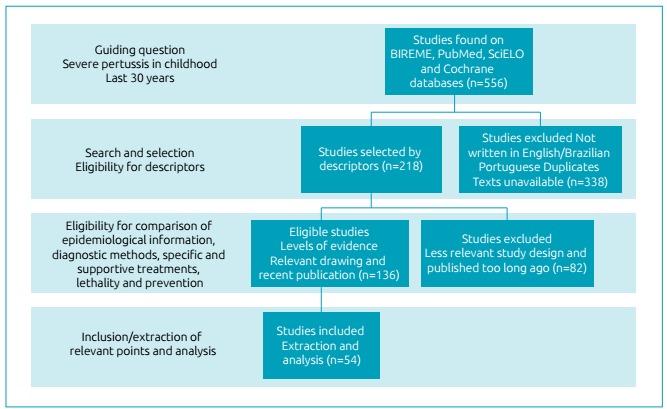
Flowchart of methods and studies selection criteria.

Systematic review of the literature carried out based on the PICO strategy (Population, Intervention, Comparison, Outcome). Historicallydefined concepts, and old and current therapeutic approaches to pertussis were used as guiding questions for bibliographic search, which was performed by two independent researchers. The descriptors “Coqueluche*/WhoopingCoughpertussis*” [All Fields] were used to search for relevant publications involving children under 18, published in the last 30years, in the US National Library of Medicine (PubMed), Scientific Electronic Library Online (SciELO), Latin American and Caribbean Literature in Health Sciences (LILACS), Cochrane and Google Scholar. Studies with levels of evidence 1A, 1B, 2A, 2B, 3A, 3B and 4, written in Brazilian Portuguese and English, protocols of the Ministry of Health (MS) and recommendations of the Centers for Disease Control and Prevention (CDC) were included. Initially, 556 papers were selected. Inclusion criteria were: studies describing epidemiology, diagnostic methods, specific and supportive treatments, lethality or prevention. After excluding duplicate records, reading full texts, and coming to an agreement between reviewers, 54 articles were kept, from which the relevant points were extracted for analysis (Figure 1).

## RESULTS AND DISCUSSION

### Epidemiological aspects

Pertussis is a disease of worldwide distribution, with epidemic cycles every three or five years.^1^ ^,^ ^5^ ^,^ ^8^ A global incidence of 16million annual cases and about 200 thousand deaths is estimated, occupying the fifth place among causes of death by immune-preventable diseases in children under five years of age.^2^ ^,^ ^6^ ^,^ ^8^ ^,^ ^9^ The incubation period lasts from 5 to 10 days and the period of transmission begins 5-7days after contact, persisting for three weeks when not treated.^2^ ^,^ ^10^ With appropriate antibiotic therapy, transmission is blocked after 5-7 days.^11^ Infectionis acquired through contact with nasopharyngeal secretions of infected persons.^3^ ^,^ ^8^ ^,^ ^12^ Asymptomatic patients are rare and not relevant in the epidemiological chain.^6^ ^,^ ^12^ ^,^ ^13^ Highly contagious pertussis has a secondary attack rate of up to 90% on susceptible intra-household contacts.^5^ ^,^ ^6^ ^,^ ^8^

In the second half of the 20th century, immunization of children with triple whole-cell bacterial vaccine, combined with tetanus and diphtheria toxoids (DPT), allowed disease control and a drastic fall in incidence rates from 200cases before 1940 to 0.5 case/100 thousand inhabitants in 2000. ^2^ ^,^ ^3^ ^,^ ^4^ ^.^ ^6^ In Brazil, incidence reduction was similar, especially since the 1990s.^1^ ^,^ ^6^ ^,^ ^12^ ^,^ ^14^ ^,^ ^15^

Although safe, the DPT vaccine causes undesirable side effects. This reactogenicity stimulated the search for new vaccines that emerged in the 1990s: DTPa (acellular triple bacterial) and dTpa (triple acellular bacterial for adolescents and adults), which have been used in developed countries.^14^ ^,^ ^16^ Immunityvaccination decreases with time: 5-14 years for DTP vaccine and 4-7 years for DTPa, depending on the age of vaccination.^4^ ^,^ ^8^ ^,^ ^14^ In Brazil, DTP is available from the National Immunization Program of the Ministry of Health, while DTPa is available at Special Immunological Reference Centers (CRIE), in specific situations and in the private network.^6^ ^.^ ^10^ ^,^ ^15^

**Figure 2:**
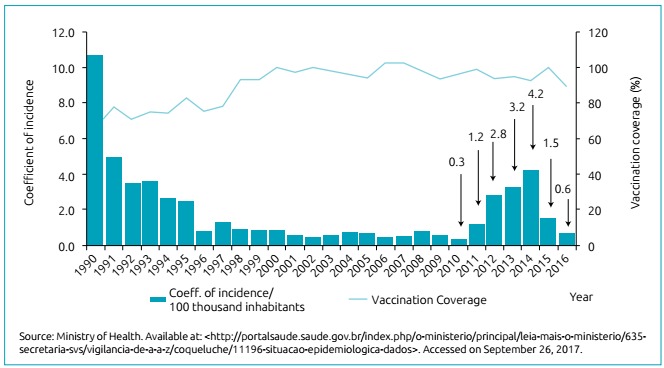
Pertussis’ coefficient of incidence and vaccine coverage. Brazil, 1990 to 2016.

As of 2000, the disease re-emerged with a modified epidemiological profile: adolescent and adult malnutrition and atypical clinical presentations, with infected mothers being the main source of transmission for their own children.^1^ ^,^ ^2^ ^,^ ^3^ ^,^ ^5^ ^,^ ^15^ ^,^ ^17^ ^,^ ^18^ Brazil has reported a similar phenomenon, although in a later time. Between 2001 and 2010, the incidence rate remained below 0.5 case/100 thousand inhabitants and, as of 2011, a progressive increase in cases was seen, reaching 4.2 cases/100 thousand inhabitants in 2014 and triggering actions of public health.^10^ ^,^ ^15^ ^,^ ^17^ ^,^ ^18^ The increase in deaths was also worrying: in Brazil: 14 deaths were recorded in 2010 and 127 in 2014, of which 97% occurred in children up to two months of age.^1^ ^,^ ^15^ ^,^ ^17^ ^,^ ^18^ This information is detailed in Figure 2.

The causes of re-emergence are not fully understood, suggesting several factors, such as genetic modifications in *Bordetella pertussis* (*B. pertussis*), with greater virulence and contagion; selection of strains not recognized by vaccines; decreased vaccination immunity, dislocation of the disease to other age groups; non-permanent protection by natural infection; less robust and lasting response by the acellular vaccine in countries recommending the use of this vaccine; and introduction of more sensitive diagnostic methods.^6^ ^,^ ^8^ ^,^ ^13^ ^,^ ^16^ ^,^ ^19^ ^,^ ^20^ ^,^ ^21^

Studies have investigated the behavior of immune response after natural infection and have concluded that the duration of immunity induced by the disease is also not permanent, remaining for 4-20 years^.^ ^9^ ^,^ ^21^ The transfer of maternal antibodies, acquired from the previous infection or childhood vaccination, is not sufficient to protect the concept, nor does lactation protect against the disease.^9^ ^,^ ^21^

Considering this new panorama, additional prevention strategies have been proposed and, in Brazil, the dTpa vaccine was incorporated into the vaccine schedule of pregnant women in 2014 aiming to induce the production of high titers of antibodies, allowing placental transfer of antibodies to the fetus, and guaranteeing protection in the first months of life.^6^ ^,^ ^15^ ^,^ ^16^

The Coccon Strategy, which consists of vaccination of adults communicating with the baby, including family members, caregivers and health professionals to form a protective “cocoon” until their immunization is completed, was partially implemented in Brazil in 2014, with vaccination of professionals directly related to neonatal care.^1^ ^,^ ^6^ ^,^ ^15^ This information is summarized in Table 1.

**Table 1:** Categorization of studies on epidemiological, microbiological and prevention aspects.

Author Country Year	Type of study Number	Relevance for inclusion	Results and conclusions
Torres SL^1^ Brazil 2015	Descriptive, cross-sectional study 1.209	Epidemiological, clinical, death and vaccination aspects	Increased incidence of pertussis and its complications
Lynfield R^2^ USA 2014	Editorial -	Review of clinical, microbiological and epidemiological aspects	Important disease in public health and reemergence in the 21st century
Cherry JD^3^ USA 2013	Editorial -	Review of clinical aspects, prevention and control	Epidemiological changes in infection and new prevention strategies
Matoo S^4^ USA 2005	Review -	Review of epidemiological, clinical and molecular biology aspects	Broad subject review, including other species of *Bordetella*
Belletini CV^5^ Brazil 2014	Retrospective study of case series 222	Clinical, laboratory and radiological predictors for pertussis	Cyanosis and lymphocytosis were independent predictors of pertussis in children up to six months of age
Zlamy M^6^ Austria 2016	Review -	Virulence factors and prevention strategies	Host-toxin interaction defines immunological vaccine modulation by natural infection
Korppi M^9^ Finland 2013	Editorial -	Review of clinical picture and prevention	Approach to disease improved over the past 50 years
SVS, MS^10^ Brazil 2014	Surveillance Protocol by Ministry of Health, Brazil -	Official protocol, with changes in definitions and criteria	Redefines case criteria and recommends preferential use of azithromycin
SVS, MS^12^ Brazil 2016	Surveillance Protocol by Ministry of Health, Brazil -	Official protocol, in use in Brazil	Update of concepts, case criteria and therapeutic recommendations
Munoz FM^13^ 2016	Review -	Pertussis in children and adolescents: diagnosis, treatment and prevention	Adolescents and adults and their importance in the chain of transmission. Recommends their immunization
McGirr A^14^ Canada 2015	Review -	Duration of vaccine immunity	Immunity conferred by DTP is longer lasting than DTPa
CGPNI, MS^15^ Brazil 2014	Technical report -	Implantation of the dTpa vaccine	Criteria and recommendations for use of the dTpa vaccine in adults
CDC^16^ 2016	CDC recommendation -	Prevention strategies	Recommendation of vaccination of the pregnant woman with dTpa
SVS, MS^17^ Brazil 2016	Epidemiological Bulletin Descriptive study 10.487	Analysis of the epidemiological situation of pertussis in Brazil, 2015	Epidemiological standard would not have changed in Brazil, continuing to undertake preferentially infants under the age of one.
SVS, MS^18^ Brazil 2015	Epidemiological Bulletin Descriptive study 72.901	Analysis of the epidemiological situation of pertussis in Brazil, 2010-2014	Increased number of cases in Brazil due to cyclical behavior of the disease
Smith AM^19^ Australia 2001	Review -	Virulence Factors	Detailed description of the virulence mechanisms
Locht C^21^ France 1999	Review -	Virulence mechanisms	Detailed description of toxins, including molecular biology

CGPNI: General Coordination of the National Immunization Program; SVS: Secretariat of Health Surveillance; MS: Ministry of Health; DTP:triple-cell whole-cell bacterial vaccine against diphtheria, pertussis and tetanus; DTPa: diphtheria, tetanus, and pertussis adsorbed vaccine; dTpa:triple acellular bacterial for use in adolescents and adults; CDC: Centers for Disease Control and Prevention.

### Clinical aspects

Pertussis is caused by the bacterium *B. pertussis*, which unlike other pathogens, does not invade tissues and bloodstream.^1^ ^,^ ^2^ Damage is caused to the respiratory tree, resulting from the production of adhesins and toxins, the most important being the pertussis toxin.^2^ ^,^ ^4^ ^,^ ^6^ ^,^ ^12^

In its classic form, it has three stages, presenting nonspecific symptoms in the first week **-** catarrhal phase **-**, resembling viral infection of the airways, with sneezing, rhinorrhea, tearing, low fever and discrete dry cough.^2^ ^,^ ^8^ ^,^ ^10^ ^,^ ^12^ From the second week **-** paroxysmal phase **-** it becomes characteristic, with episodes of paroxysmal coughing and cramps, which can last for two to six weeks.^4^ ^,^ ^8^ At this stage, lymphocytosis appears, with above 20,000 leukocytes/mm^3^, although it may be absent in partially immunized patients or when secondary bacterial infection occurs.^4^ ^,^ ^8^ In the third phase **-** convalescence **-** progressive decrease in the number and severity of cough attacks is observed, lasting for up to four weeks.^2^ ^,^ ^8^ ^,^ ^16^ ^.^ ^22^

Factors such as age, immunological status, strain virulence, vaccine status and time elapsed from vaccination impact clinical presentation.^4^ ^,^ ^13^ ^,^ ^23^ Soft clinical manifestations are common in partially immunized children and adolescents and adults with evanescent immunity, in whom only prolonged dry cough, for more than 21 days is reported, leading to diagnostic confusion with bronchitis, sinusitis, atypical pneumonia and allergies.^2^ ^,^ ^8^ ^,^ ^14^ ^,^ ^23^ ^,^ ^24^ Another diagnostic difficulty refers to newborns, whose clinical picture may present only with cough and cyanosis, without paroxysms or chirping.^7^ ^,^ ^8^ The overlapping of bacterial infections and viral coinfections, especially by Adenovirus, Respiratory Syncytial Virus, Parainfluenza, Metapneumovirus and Bocavirus, alters clinical and laboratorial evolution, interfering in diagnosis and treatment, and increasing hospitalization time and complications.^3^ ^,^ ^5^ ^,^ ^8^ Co-infections occur from immunologic depression caused by pertussis toxin, favoring the establishment of secondary pathogens.^5^

Classical, uncomplicated pertussis presents without high fever.^10^ ^,^ ^16^ Complications are seen in the paroxysmal phase, including cyanosis and apnea, edema, facial congestion and petechia arising from cough, otitis media caused by *B. pertussis* or secondary infection, and dehydration, vomiting, or low ingestion.^14^ ^,^ ^16^ ^,^ ^25^ ^,^ ^26^ Among respiratory complications are atelectasis, *B. pertussis* pneumonia and viral or bacterial superinfection. Neurological complications are less frequent and include seizures, blindness, and deafness.^25^ ^,^ ^27^ Kazantziet al. described characteristics of 31patients with pertussis admitted to six ICUs over an 11-year period, highlighting hyponatremia (serum sodium between 125-133mmol/dL) secondary to inappropriate secretion of antidiuretic hormone as a risk factor for death, which had not been described so far.^28^

In the 2000s, the concept of malignant pertussis was established, being characterized by severe and high lethality, defined by the presence of acute respiratory failure, pulmonary hypertension and hyperleukocytosis above 50,000/mm^3^.^25^ ^,^ ^29^ Hyperleukocytosis causes blood hyper-viscosity and increases pulmonary vascular resistance, leading to pulmonary hypertension and hemodynamic collapse, with death due to hypoxemia and refractory shock.^29^ ^,^ ^30^ Paddock et al. evaluated lung tissues of 15 infants with death due to pertussis, demonstrating the presence of necrotizing bronchiolitis, intra-alveolar hemorrhage and *B. pertussis* inside the bronchioles and alveoli.^26^ ^,^ ^30^ Similarly,Palvo et al., in 2017, described the necropsy process of six young and unvaccinated infants, all presenting with thickening of the pulmonary arterioles, pulmonary hypertension, which points to co-infection by *B. pertussis* and Respiratory Syncytial Virus in the same tissues.^31^ Predictivefactors of severity and risk of death include age less than six months, hyperleukocytosis, pulmonary hypertension and the presence of comorbidities.^29^ ^,^ ^31^ ^,^ ^32^ ^,^ ^33^ Palvo et al. reported a cutoff point of 41,000 leukocytes/mm^3^ to predict ICU admission, with sensitivity of 65% and specificity of 90%; and sensitivity of 100% and specificity of 81.6% to predict death.^31^

### Diagnosis

The suspicion and confirmation criteria for pertussis were updated by the Ministry of Health in 2014, following the CDC criteria, modified in 2005.^10^ ^,^ ^16^ The main changes refer to the inclusion of RT-PCR (real-time polymerase chain reaction), reduction of coughing time from 14 to 10 days, subdivision at ages below and above six months, and withdrawal of lymphocytosis as confirmation criterion.^10^ Lymphocytosis arises from the second week of disease on, and may be absent in partially immunized patients.^3^ In addition, intercurrent bacterial infections occur with neutrophilia, which results in confusion. Therefore, the absence of lymphocytosis does not exclude the diagnosis of pertussis.^5^ ^,^ ^10^

Etiological diagnosis can be made by microbiological, immunological and molecular exams.^4^ ^,^ ^33^ ^,^ ^34^ The isolation of *B. pertussis* in the culture of deep nasopharyngeal secretion is gold-standard due to its high specificity.^4^ ^,^ ^12^ ^,^ ^35^ ^,^ ^36^ However, its sensitivity varies (12-28%) and depends on the conditions of collection, storage, transport and incubation of the sample, as well as disease stage, previous use of antimicrobials and number of vaccine doses received.^36^ ^,^ ^37^ The development of RT-PCR allowed greater sensitivity (up to 72%) and rapid diagnosis, without requiring the presence of viable bacteria, with positive results after the second week of disease even in vaccinated individuals with prior antibiotic use.^34^ ^,^ ^38^ ^,^ ^39^

Although the immunologic diagnosis is not standardized in Brazil, it is used in other countries.^10^ Methods available include detection of immunoglobulin G (IgG) against filamentous toxin (Centers for Disease Control and Prevention**-** CDC, Pertussis EnzymeLinked ImmunoSorbent Assay **-** PT-ELISA) and fluorescent antibody (DFA) screening. These have little practical application, since antibodies are late detectable, and transplacental passage of antibodies and previous vaccination may interfere with the results.^34^ Itis useful symptoms persist beyond three weeks, with analysis of the appearance or increase of IgG in two samples collected with an interval of 14 days.^34^ The selected articles referring to the clinical and diagnostic aspects are summarized in Table 2.

**Table 2:** Categorization of selected studies with approach to clinical and diagnostic aspects.

Author Country Year	Type of study Number	Relevance for inclusion	Results and conclusions
Heininger U^22^ Germany 1997	Cohort 20.972	Clinical and laboratory aspects	Classical and laboratory clinical picture in non-vaccinated patients. Pneumonia was the most frequent complication.
Yildirim I^23^ Turkey 2008	Cohort 148	Clinical and laboratory aspects	Clinical presentation was not always typical
SVS, MS^24^ Brazil 2009	Surveillance Protocol by Ministry of Health, Brazil	Official protocol, with pre-updated definitions and criteria	Case criteria and therapeutic recommendations
Elliot E^27^ Australia 2004	Descriptive study 140	Severe pertussis	Pertussis as an important cause of morbidity and mortality
Kazantzi M^28^ Greece 2017	Multicenter descriptive study	Characteristics and Complications of pertussis	Higher mortality in young infants Hyperleukocytosis, mechanical ventilation and hyponatremia are associated with higher lethality
Bouziri A^29^ Tunisia 2010	Descriptive study 10	Malignant pertussis	Malignant pertussis is often underdiagnosed and fatal in infants less than three months old
Paddock CD^30^ USA 2008	Observational anatomopathological study 15	Severe pertussis	Necropsy in pulmonary tissue with hypertension and presence of *B. pertussis* in the lung
Palvo F^31^ Brazil 2017	Observational anatomopathological study 6	Severe pertussis	Necropsy in lung tissue with pulmonary hypertension, respiratory syncytial virus and *B. pertussis* in lungs and kidneys
Milekova^32^ Canada 2003	Case-control study 48	Severe pertussis	Leukocytosis and pneumonia were death predictors in infants less than two months old
Marshall H^33^ Australia 2015	Cohort 120	Severe pertussis	Presence of co-infections, prematurity and high fever require rigorous monitoring
Vaz de Lima^34^ Brazil 2014	Experimental study 503	Laboratory diagnosis	Serology as an auxiliary method in late diagnosis
Regan J^35^ Canada 1977	Experimental study 3.237	Laboratory diagnosis	Description of gold-standard method for *B. pertussis* culture
Gilligan PH^36^ USA 1984	Experimental study 223	Laboratory diagnosis	Culture was more sensitive than serology for diagnosis of *B. pertussis*
Müller FM^37^ Germany 1997	Review	Laboratory diagnosis	Review of the laboratory diagnostic method. Introduction of the PCR exam in the laboratory routine
Inst. Ad. Lutz^38^ Brazil 2010	Manual of laboratory diagnosis	Laboratory diagnosis	Describes standards, routines and recommendations by the national reference laboratory
Reish U^39^ Germany 2001	Experimental study 113	Laboratory diagnosis	RT-PCR technique showed high specificity and high predictive value
Lopez MA^48^ USA 2014	Cohort 1.012	Severe pertussis	Neonates and children with chronic diseases are the most vulnerable groups, requiring hospitalization

SVS: Secretariat of Health Surveillance; MS: Ministry of Health; PCR: polymerase chain reaction; RT-PCR: real-time polymerase chain reaction.

### Therapeutic approach

####  Antibiotic therapy 

The literature is unanimous in stating that patients with suspected pertussis should receive antibiotic therapy prior to diagnostic confirmation.^11^ ^,^ ^12^ ^,^ ^40^ ^,^ ^41^ ^,^ ^42^ In the catarrhal stage, antibiotic therapy may reduce the severity of symptoms and the duration of the disease, as well as accelerate the elimination of bacteria in the nasopharynx.^11^ ^,^ ^12^ ^,^ ^40^ ^,^ ^41^ In the paroxysmal phase, it is indicated to reduce transmissibility, eliminating nasopharyngeal bacteria after 5-7 days from the beginning of treatment.^11^ ^,^ ^42^ ^,^ ^43^ Macrolides are the first and all studies found before 1996 recommended the use of erythromycin estolate at a dose of 40 mg/kg/day, each 6 hours for 7-14 days.^41^ From1990, studies have investigated the minimum time necessary for eradication of *B. pertussis*, evaluating whether diagrams of seven or ten days would be sufficient and whether the interval between doses could be 8 hours.^26^ ^,^ ^42^ ^,^ ^43^ In the 1990s, resistance of *B. pertussis* to erythromycin was described in some countries, which led to the search for new therapeutic options along with adverse gastrointestinal effects such as nausea, vomiting and diarrhea.^4^ ^,^ ^41^ ^,^ ^42^ In this review, no studies on erythromycin resistance were found in Brazil and international reviews do nor report concern.^4^ ^,^ ^8^

Studies performed from 2000 have evaluated the efficacy and safety of azithromycin and clarithromycin.^43^ Astudy by Langley et al., in 2004, followed-up 477 children and adolescents who received azithromycin for five days (10 mg/kg once daily in the first day and 5 mg/kg once daily for another four days) and erythromycin estolate 40 mg/kg/day each 8hours for 10 days.^43^ Another study conducted by Lebel in 2001 followed 153 children and adolescents who received clarithromycin (7.5mg/kg/dose, each 12 hours for seven days) and erythromycin (13mg/kg/dose, each 8hours, for 14 days).^44^ ^,^ ^45^ Both studies concluded that the therapeutic regimens had similar efficacy, with reduction of gastrointestinal adverse effects in the groups receiving azithromycin and clarithromycin. The last systematic review on pertussis antibiotic therapy was published in 2007 and analyzed 13studies, concluding that short treatments with azithromycin for three to five days and with clarithromycin or erythromycin for seven days were just as effective for the eradication of nasopharyngeal bacteria when compared to longer treatments, with less adverse effects.^40^ More recently, Dierig et al., in 2015, reported two children who had persistence of *B. pertussis* in the nasopharynx after seven days of treatment with clarithromycin.^11^ Thus, the ideal minimum time required is still under questioning.

Regarding safety, we highlight hypertrophic pyloric stenosis as an adverse event of macrolides in infants less than six months of age.^46^ In a multicenter study with 999,378 children exposed to macrolides from gestation up to 120 days of life, Lund etal., in 2014, concluded that exposure to macrolides, especially erythromycin, in the first weeks of life, increases the chance of hypertrophic pyloric stenosis up to 30 times.^46^ Another controversial aspect is the age-dependent clinical response, since macrolides alleviates symptoms of pertussis in infants, but not in older children.^9^ The different clinical responses seem to be related to the duration of infection.^8^ ^,^ ^39^ When diagnosis and treatment are delayed, the already established damage reduces the action of the antibiotic.^8^ ^,^ ^29^ ^.^ ^40^

**Figure 3:**
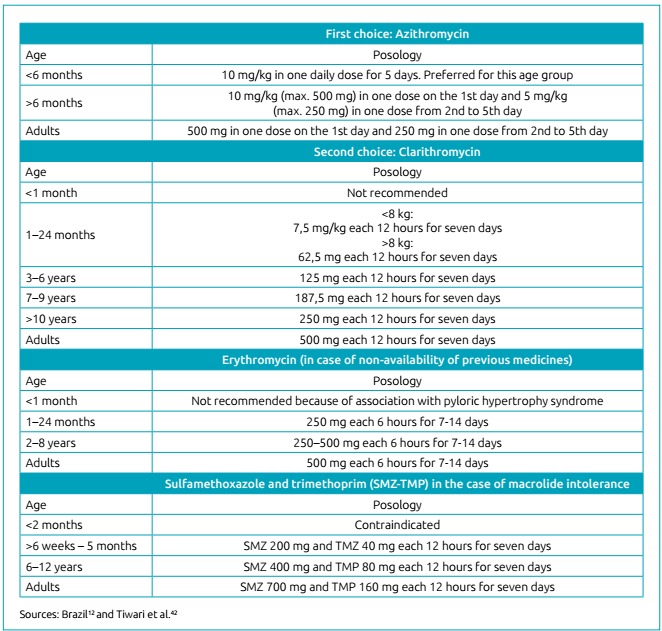
Antibiotic therapy and chemoprophylaxis for pertussis.

In Brazil, the Ministry of Health started to recommend azithromycin as a choice in the treatment and chemoprophylaxis of pertussis as of 2014.^10^ Clarithromycin became the second option, with restriction of use in children less than one month old, while erythromycin should only be prescribed if the other macrolides are unavailable.^10^ In the same protocol, sulfamethoxazole-trimethoprim remains as a therapeutic option in cases of macrolide intolerance, and is still contraindicated in children less than two months of age.^10^ Ampicillin, cephalosporins and fluoroquinolones have not shown effectiveness required to eliminate *B. pertussis*.^8^ ^,^ ^42^ The current schemes recommended by the Ministry of Health and CDC are summarized in Figure 3.

####  Hospital support for severe pertussis 

In addition to specific antibiotic therapy, severe cases of pertussis require additional approaches to advanced life support to minimize complications.^47^ ^,^ ^48^ Hospitalization is determined by the risk of serious progression, and newborns and young infants should be considered as patients with pulmonary, muscular or neurological disease, in which complications are more frequent.^6^ ^,^ ^8^ ^,^ ^25^ ^,^ ^29^ ^,^ ^47^ ^,^ ^48^ Adequatehydration is essential for the fluidification of respiratory secretions and maintenance of blood volume.^12^ ^,^ ^48^ Oxygentherapy is more frequently needed due to apnea, respiratory distress from pneumonia, intercurrent viral and bacterial infections, and pulmonary hypertension, being recommended in paroxysms and cyanosis crises.^8^ ^,^ ^12^ ^,^ ^47^ ^,^ ^48^ Infantswhose fatigue results in hypercapnia are indicated for mechanical ventilation.^6^ ^,^ ^8^ ^,^ ^25^ ^,^ ^48^

Initial signs of alarm are: tachypnea (respiratory rate above 60 respiratory incursions per minute); persistent hypoxia after paroxysms; leukocytosis above 50,000 cells/mm^3^; and heart rate below 60 beats per minute.^29^ ^,^ ^47^ ^,^ ^48^ In a prospective multicenter study, Berger et al. correlated hyperleukocytosis with a tenfold increased risk of death.^25^ High predictors of lethality and/or neurological sequelae are: apnea and bradycardia during episodes of cough; change in mental state; seizures; less than six months of age, especially less than two months of age; associated pneumonia; presence of comorbidities; and shock, with bradycardia in episodes of cough, seizures and pulmonary hypertension as isolated predictive factors for death.^25^ ^,^ ^30^ ^,^ ^31^ ^,^ ^47^ ^,^ ^48^

####  Adjuvant treatment of malignant pertussis 

Adjuvant treatment of malignant pertussis is still controversial.^49^ ^,^ ^50^ ^,^ ^51^ In a case report, Romano et al. described successful treatment of patients with severe pertussis, respiratory failure and hyperleukocytosis, with white blood cell count of 104,000/mm^3^ and pulmonary hypertension, submitted to exchange transfusion and showing rapid reduction in the leukocyte mass.^49^ However, in 2013, Nieves et al. reported ten cases with conflicting results and recommended caution.^50^ Leukapheresis therapy has been used since 1990 to reduce leukocyte mass, considering that hyperleukocytosis and blood hyper-viscosity are responsible for severe pulmonary involvement.^49^ ^,^ ^52^ In a follow-up study of 19 patients submitted to leukapheresis between 2001 and 2009, Rowlands et al. pointed out that this procedure may contribute to the survival of critically affected patients, but its use is limited by serious adverse effects and high cost.^50^ ^,^ ^51^ ^,^ ^52^

A multicenter study by Berger et al. Evaluated127patients up to 18 years of age with confirmed pertussis, 83% of whom were less than three months of age.^25^ Of participants, 43% required mechanical ventilation and 9.4% died. All 16 cases (13%) with pulmonary hypertension required mechanical ventilation and 14 received nitric oxide. Of death cases, 75% were due to pulmonary hypertension. Leukocytosis was more relevant among patients who required mechanical ventilation, had pulmonary hypertension or died. Amongall patients in the study, 14 (11%) had hyperleukocytosis and received therapies to reduce leukocyte mass: 12 received exchange transfusion, one leukapheresis and the other patient received both treatments.^25^ In 2010, Bouziri et al. also concluded that hyperleukocytosis is associated with the need for mechanical ventilation, pulmonary hypertension and increased risk of death, but they pointed out the need for further studies to clarify the real benefits of these therapies, as they bring serious adverse effects.^29^

Since 1990, other therapies have been described, such as Extracorporeal Membrane Oxygenation (ECMO), in patients with severe-pertussis respiratory failure, but there is no consensus on its efficacy.^7^ ^,^ ^53^ Potential therapies still in experimental phase were used by Scanlon et al., including immunosuppressants and anion channel modulators such as Pendrine, Acetazolamide, and Fingolimod; some of these have been proposed for cystic fibrosis, tuberculosis and autoimmune diseases and proven beneficial in animal models.^7^ The authors are currently awaiting regulatory approval for testings in humans.^7^

####  Symptomatic treatment of cough 

Several symptomatic treatments for pertussis cough, including corticosteroids, salbutamol, anti-pertussis immunoglobulin, antihistamines and leukotriene inhibitors are well known.^7^ ^,^ ^12^ Since 1970, corticosteroids have been used to reduce paroxysms, as they were believed to alter the severity and course of the disease. The recommendation consisted in using full-dose hydrocortisone for two days followed by progressive reduction with suspension in five to six days.^7^ ^,^ ^8^ Thisstrategy was abandoned due to lack of efficacy evidence.^4^ ^,^ ^8^ Humananti-pertussis immunoglobulin had been used in the last decades, but was abandoned due to lack of proven therapeutic value.^7^ ^,^ ^8^ Anticonvulsants have been used not only to treat seizures, but also as sedatives, reducing paroxysmal intensity. However, their use was abandoned due to lack of evidence.^7^ ^,^ ^8^ ^,^ ^25^ ^,^ ^54^ Bronchodilators are still prescribed, even though they are not very effective, especially salbutamol.^7^ ^,^ ^25^ ^,^ ^54^

Cochrane’s latest review of pertussis in 2014 evaluated the effectiveness and safety of interventions to reduce paroxysms.^54^ Twelve studies were included and had the following results reported: diphenhydramine did not alter cough episodes; anti-pertussis immunoglobulin led to a reduction in coughing time in one day, without shortening hospital stay; dexamethasone did not reduce hospitalization rate; Salbutamol also did not change the course of paroxysms; and montelukast led to a decrease in the number of cough accesses per day, without clinical and statistical significance. Thus, the authors concluded that there is insufficient evidence to recommend such interventions and their use should be discouraged.^54^ Studies on therapeutic approach to cough (antibiotic therapy, hospital support, adjunctive treatment, and symptomatic treatment) are listed and summarized in Table 3.

**Table 3:** Categorization of studies on therapeutic approach.

Author Country Year	Type of study Number	Relevance for inclusion	Results and conclusions
Scanlon KM^7^ USA 2015	Review -	New potential treatments for pertussis	Clinical studies needed to evaluate effectiveness of Pendrin and Acetazolamide
Kilgore PE^8^ USA 2016	Review -	Review of microbiology, clinical aspects, treatment and prevention	Comprehensive review on pertussis
Dierig A^11^ Switzerland 2015	Case report 2	Duration of treatment with clarithromycin	Positive PCR tests after seven days of clarithromycin
Wood N^20^ Australia 2008	Review -	Review of epidemiology, diagnosis, treatment and prevention	Broad review
Berger JT^25^ USA 2013	Cohort 127	Severe pertussis: supportive treatment	Hyperleukocytosis reduced by Nitric oxide Exchange transfusion indicated for pulmonary hypertension
Halperin SA^26^ Canada 1997	Controlled, randomized, double blind study 168	Time of erythromycin use for bacteria eradication	Seven days of erythromycin as effective as 14 days for bacterial eradication in the nasopharynx
Altunaiji S^40^ USA 2007	Systematic review 13	Treatment and prophylaxis of pertussis	All macrolides eradicate bacteria but do not alter the course of the disease
Bass JW^41^ Hawaii 1986	Controlled, double-blind, randomized clinical trial 50	Classical study: use of erythromycin for treatment and prevention	Erythromycin more effective than other antibiotics for bacterial eradication
Tiwari T, CDC^42^ USA 2005	Recommendation	Antibiotic therapy and chemoprophylaxis	Recommends replacement of erythromycin with azithromycin
Langley JM^43^ Canada 2004	Randomized, double blind clinical study 477	Azithromycin and erythromycin Eradication of bacteria, clinical and adverse effects	Seven days of azithromycin as effective as 14 days of erythromycin, with fewer adverse effects
Korgenski^44^ USA 1997	Experimental study 47	Resistance of B. pertussis to erythromycin	Resistance of *B. pertussis* to erythromycin was uncommon (1985 to 1997)
Lebel MH^45^ USA 2001	Randomized, single blind study 153	Clarithromycin and azithromycin: efficacy and safety	Clarithromycin as effective as erythromycin with fewer side effects
Lund M^46^ USA 2014	Multicenter cohort study 999,378	Hypertrophic pyloric stenosis as an adverse effect of macrolides	Use of macrolides in neonates increased the risk of hypertrophic pyloric stenosis
Surridge J^47^ New Zealand 2007	Cohort 72	Severe pertussis: clinic and severity criteria	Apnea and early paroxysms (less than a week of symptoms) are signs of severity and require ICU admission
Romano MJ^49^ USA 2004	Case report 1	Severe pertussis: supportive treatment	Exchange transfusion effective for leukoreduction
Nieves D^50^ Canada 2013	Descriptive study10	Severe pertussis: supportive treatment	Exchange transfusion effective if performed early, before organ failure
Rowlands HE^51^ England 2010	Descriptive study19	Severe pertussis: supportive treatment	Leukoreduction therapies may be considered safe in critically ill patients
Grzeszczak MJ^52^ USA 2006	Case report 1	Severe pertussis: supportive treatment	There was success in treatment with leukopheresis
Halasa NB^53^ USA 2003	Case report 4	Severe pertussis: supportive treatment	Use of ECMO was controversial. All patients died.
Wang K^54^ USA 2014	Systematic review 10	Symptomatic treatment of pertussis	No symptomatic treatment of cough was effective

PCR: polymerase chain reaction; ICU: Intensive Care Unit; ECMO: Extracorporeal Membrane Oxygenation.

## CONCLUSION

The approach to severe pertussis in childhood remains a challenge. Much progress has been made in recent years when it comes to syndromic and etiological diagnosis of pertussis, especially with the introduction of RT-PCR as diagnostic method. However, the therapeutic options currently available are still unsatisfactory. The replacement of erythromycin with azithromycin made treatment easier but, although it was effective in interrupting transmission, its ability to change the course of the disease is timid, especially when treatment is delayed and there are still queries about the best therapeutic regimen, especially in severe cases. In addition, coughing lasts for months, as no symptomatic medication has shown efficacy. ICU support treatment improved the prognosis of patients with respiratory failure and pulmonary hypertension, mainly with mechanical ventilation and nitric oxide, but further studies are needed to determine the role of adjuvant therapies. As for procedures for leukocyte reduction, plasmapheresis has high cost and serious adverse effects, so its indication is controversial, unlike exchange transfusion, which has been shown to be effective for malignant pertussis. Studies involving other therapies for modulation of immune response, such as the use of Acetazolamide and Pendrine, have shown promising in the experimental phase, thus requiring confirmation of efficacy and safety in future clinical studies.
